# Due-Window Assignment Scheduling with Variable Job Processing Times

**DOI:** 10.1155/2015/740750

**Published:** 2015-03-30

**Authors:** Yu-Bin Wu, Ping Ji

**Affiliations:** ^1^School of Science, Shenyang Aerospace University, Shenyang 110136, China; ^2^Department of Industrial and Systems Engineering, The Hong Kong Polytechnic University, Hung Hom, Kowloon, Hong Kong

## Abstract

We consider a common due-window assignment scheduling problem jobs with variable job processing times on a single machine, where the processing time of a job is a function of its position in a sequence (i.e., learning effect) or its starting time (i.e., deteriorating effect). The problem is to determine the optimal due-windows, and the processing sequence simultaneously to minimize a cost function includes earliness, tardiness, the window location, window size, and weighted number of tardy jobs. We prove that the problem can be solved in polynomial time.

## 1. Introduction

In most scheduling studies, job processing times are treated as constant numbers; however, in many practical situations, job processing times are affected by the learning effects and/or deteriorating (aging) effects. Learning effects and deteriorating (aging) effects are important for production and scheduling problems. For details on this line of the scheduling problems with learning effects (deteriorating effects), the reader is referred to a comprehensive survey by Biskup [1] (Gawiejnowicz [2]). Rudek [3] considered single machine scheduling problems with position-dependent job processing times (i.e., learning and aging effects). For the following objectives, the makespan with release dates, the maximum lateness, and the number of late jobs, they gave some results. J.-B. Wang and M.-Z. Wang [4] and Sun et al. [5] considered flow shop scheduling problems with general position-dependent learning effects. For some regular objective functions, they proposed heuristics. Sun et al. [6] considered flow shop scheduling problems with three special position-dependent learning effects. For the total weighted completion time minimization problem, they proposed heuristics. Lu et al. [7] considered single machine scheduling problems with learning effects and controllable processing times. For two due date assignment methods, they presented a polynomial-time optimization algorithm to minimize a multiobjective cost function.

J.-B. Wang and M.-Z. Wang [8] considered common due-window single machine scheduling with learning effects and controllable processing times. For a mule-objective cost function, they presented a polynomial-time optimization algorithm. J.-B. Wang and M.-Z. Wang [9] considered single machine scheduling problems with nonlinear deterioration. They showed that the makespan minimization problem can be solved in polynomial time. J.-B. Wang and M.-Z. Wang [10] considered three-machine flow shop scheduling with deteriorating jobs. For the makespan minimization problem, they proposed a branch-and-bound algorithm and two heuristic algorithms. X.-R Wang and J.-J. Wang [11] considered single machine scheduling problems with deteriorating jobs and convex resource dependent processing times. Xu et al. [12] considered single machine group scheduling with proportional linear deterioration and ready times. For the makespan minimization problem, they gave some results. Cheng et al. [13] considered a single machine common due-window assignment scheduling problem with deteriorating jobs. For a deteriorating maintenance activity, they provided polynomial-time solutions for a multiobjective cost. Yang et al. [14] considered a single machine multiple common due dates assignment resource allocation scheduling problems with general position-dependent deterioration effect. For a multiobjective cost, they proved that the problems can be solved in polynomial time, respectively. Liu et al. [15] considered single-machine common due-window assignment scheduling problem with deteriorating jobs. If the width of the common due-window is a given constant, they proved a mule-objective function cost problem can be solved in polynomial time. J.-B. Wang and C. Wang [16] and Wang et al. [17] considered due-window assignment scheduling problems with learning effects and deteriorating jobs at the same time.

The recent paper Li et al. [18] addresses single machine scheduling problem with deteriorating jobs. For common due date assignment (CON) and common flow allowance (i.e., all jobs have slack due date (SLK)) due date assignment methods, they showed that a multiobjective minimization problem can be solved in polynomial time, respectively. In this research, we continue the work of Li et al. [18] but focus on the common due-window assignment (CONW) scheduling problem (Yin et al. [19]). Under the learning effect and deteriorating jobs models, we prove that the CONW due-window assignment scheduling is solvable in polynomial time, respectively.

## 2. Problem Formulation

The following notations will be used throughout the paper:

 
*J*_*j*_: Job *j*
 
*J*: Set of jobs (i.e., *J* = {*J*_1_, *J*_2_,…, *J*_*n*_})
 
*C*_*j*_: Completion time of job *J*_*j*_
 
*d*_1_: Earliest due date
 
*D*: Common due-window size
 
*d*_2_: Latest due date = *d*_1_ + *D*
 
*E*_*j*_: Earliness of *J*_*j*_ = max⁡{0, *d*_1_ − *C*_*j*_}
 
*T*_*j*_: Tardiness of *J*_*j*_ = max⁡{0, *C*_*j*_ − *d*_2_}
 
*E*: Set of earliest jobs = {*J*_*j*_∣*C*_*j*_ < *d*_1_}
 
*T*: Set of tardy jobs = {*J*_*j*_∣*C*_*j*_ > *d*_2_}
 
$\overset{-}{D}$: Set of on time jobs (i.e., $\overset{-}{D}=J\setminus (E\cup T)$)
 
*m*: Number of set $\overset{-}{D}$ jobs (i.e., $m=|\overset{-}{D}|$)
 
*γ*_*j*_: The penalty weight if *J*_*j*_ is tardy (i.e., *J*_*j*_ ∈ *T*)
 
*F*(*d*_1_, *D*, *π*) = *αd*_1_ + *βD* + *θ*∑_*J*_*j*_∈*E*_ *E*_*j*_ + ∑_*J*_*j*_∈*T*_ *γ*_*j*_: The total cost function, where *α* > 0, *β* > 0, and *θ* > 0 are the unit due-window starting time, due-window size, and earliness penalties, respectively.

Consider a nonpreemptive single machine setting. There are* n* independent jobs *J* = {*J*_1_, *J*_2_,…, *J*_*n*_} available at zero and preemption is not allowed. Let *P*_*j*_ denote the actual processing time for job *J*_*j*_. In this research, we consider the following models.

*Job Time-Dependent Deterioration Effect Model (See Li et al. [18])*. Consider

(1) $$\begin{array}{c} P_{j}=a_{j}+bt, \end{array}$$

where *a*_*j*_, *b* > 0, *t* are the basic (normal) processing time of *J*_*j*_, the deteriorating rate, and the starting time of *J*_*j*_, respectively.

*Job-Position-Dependent Learning Effect Model (See Biskup [20])*. Consider

(2) $$\begin{array}{c} P_{j}=a_{j}r^{a}, \end{array}$$

where *a*_*j*_, *a* < 0, *r* are the basic (normal) processing time of *J*_*j*_, the learning rate, and the position *J*_*j*_ in a processing sequence, respectively.

Our task of this paper is to determine the optimal earliest due date *d*_1_, the common due-window size *D*, and a schedule *π* which minimizes the following objective function:

(3) $$\begin{array}{c} F\left(d_{1},D,\pi \right)=\alpha d_{1}+\beta D+\theta \underset{J_{j}\in E}{\sum }E_{j}+\underset{J_{j}\in T}{\sum }\gamma_{j}. \end{array}$$

Then, using the common three-field notation introduced by Graham et al. [21], the corresponding scheduling problems are denoted by

(4) $$\begin{array}{c} 1\;|\;P_{j}=a_{j}+bt\;|\;\alpha d_{1}+\beta D+\theta \underset{J_{j}\in E}{\sum }E_{j}+\underset{J_{j}\in T}{\sum }\gamma_{j},\\ 1\;|\;P_{j}=a_{j}r^{a}\;|\;\alpha d_{1}+\beta D+\theta \underset{J_{j}\in E}{\sum }E_{j}+\underset{J_{j}\in T}{\sum }\gamma_{j}. \end{array}$$

## 3. Optimal Solutions

### 3.1. Job Time-Dependent Deterioration Effect Model

**Lemma 1 (Li et al. [18]).**

For a given schedule *π* = (*J*_[1]_, *J*_[2]_,…, *J*_[*n*]_), if the starting time of the first job is 0, then *C*_[*r*]_ = ∑_*j*=1_^*r*^ *a*_[*j*]_(1 + *b*)^*r*−*j*^ and ∑_*j*=1_^*n*^ *C*_*j*_ = ∑_*j*=1_^*n*^ *a*_[*j*]_∑_*i*=0_^*n*−*j*^(1 + *b*)^*i*^.

**Lemma 2 .**

If *α* > *β*, an optimal schedule exists in which the due-window starts at time zero.

**Proof**

Suppose *α* > *β*, and *d*_1_ > 0; we shift *X* units of time to the left. The change in the total cost is given by Δ*Z* = −*αX* + *βX* − *θlX*, where *l* denotes the number of early jobs. Cleary, Δ*Z* < 0. Therefore, a shift of *d*_1_ (until *d*_1_ = 0) can only decrease the total cost.

**Lemma 3 .**

An optimal schedule exists in which the due-window starting time (i.e., *d*_1_), and the due-window completion time (i.e., *d*_2_) coincide with job completion times, respectively.

**Proof**

Suppose that there exists a schedule starting at time zero and containing jobs at the *k*th and the (*k* + *m*)th positions such that *C*_*k*_ < *d*_1_ < *C*_*k*+1_, *C*_*k*+*m*_ < *d*_2_ < *C*_*k*+*m*+1_.

 When we shift *d*_2_ to *C*_*k*+*m*_, the change in the total cost is given by −*β*(*d*_2_ − *C*_*k*+*m*_).
 When we shift *d*_1_ to *C*_*k*_, the change in the total cost is given by (−*α* + *β* + *kθ*)(*d*_1_ − *C*_*k*_).
 When we shift *d*_1_ to *C*_*k*+1_, the change in the total cost is given by −(−*α* + *β* + *kθ*)(*C*_*k*+1_ − *d*_1_).
 Again, a shift of *d*_1_ to *C*_*k*_ or to *C*_*k*+1_ does not increase the total cost.

Therefore, an optimal schedule exists such that both *d*_1_ and *d*_2_ coincide with job completion times.

**Lemma 4 .**

An optimal schedule exists in which the index of the job completed at the due-window starting time is *k* = ⌈(*β* − *α*)/*θ*⌉.

**Proof**

Using the classical small perturbation technique (see J.-B. Wang and C. Wang [16] and J.-B. Wang and M.-Z. Wang [8]), we measure the change in the total cost when moving *d*_1_.

We shift *d*_1_, *X* units of time to the left, and the effect of the total cost is

(5) $$\begin{array}{c} -\alpha X+\beta X-\theta \left(k-1\right)X. \end{array}$$

We shift *d*_1_, *X* units of time to the right, and the effect of the total cost is

(6) $$\begin{array}{c} \alpha X-\beta X+\theta kX. \end{array}$$

Both expressions (5) and (6) are clearly nonnegative due to the optimality of the original solution.

From −*αX* + *βX* − *θ*(*k* − 1)*X* ≥ 0 and *αX* − *βX* + *θkX* ≥ 0 we have *k* ≤ ((*β* − *α*)/*θ*) + 1 and *k* ≥ (*β* − *α*)/*θ*. And from the integrality of* k*, it follows that *k* = ⌈(*β* − *α*)/*θ*⌉.

**Lemma 5 .**

For the problem 1∣*P*_*j*_ = *a*_*j*_ + *bt*∣*αd*_1_ + *βD* + *θ*∑_*J*_*j*_∈*E*_ *E*_*j*_ + ∑_*J*_*j*_∈*T*_ *γ*_*j*_, if the job sequence is *π* = (*J*_[1]_, *J*_[2]_,…, *J*_[*n*]_) and $m=|\overset{-}{D}|$, then the objective function can be expressed as

(7) $$\begin{array}{c} F\left(d_{1},D,\pi ,m\right)=\overset{k+m}{\underset{j=1}{\sum }}w_{j}a_{\left[j\right]}+\overset{n}{\underset{j=k+m+1}{\sum }}\gamma_{\left[j\right]}, \end{array}$$

where

(8) $$\begin{array}{c} w_{j}=\left\{\begin{array}{cc} \left\{\left(\alpha +k\theta \right)+\beta \left[{\left(1+b\right)}^{m}-1\right]\right\}{\left(1+b\right)}^{k-j} & \\ \;-\theta \overset{k-j}{\underset{i=0}{\sum }}{\left(1+b\right)}^{i},\;\;j=1,2,\ldots ,k; & \\ \beta {\left(1+b\right)}^{k+m-j},\;\;\;j=k+1,k+2,\ldots ,k+m. & \end{array}\right. \end{array}$$

**Proof**

By Lemmas 1 and 3, we have

(9)d1=C[k]=∑j=1ka[j]1+bk−j,(10)D=Ck+m−Ck=∑j=1k+ma[j]1+bk+m−j−∑j=1ka[j]1+bk−j=∑j=1kaj1+bk+m−j+∑j=k+1k+maj1+bk+m−j −∑j=1ka[j]1+bk−j=∑j=1kaj1+bk−j1+bm+∑j=k+1k+maj1+bk+m−j −∑j=1ka[j]1+bk−j=∑j=1kaj1+bk−j[1+bm−1] +∑j=k+1k+maj1+bk+m−j,Fd1,D,π,m =αd1+βD+θ∑Jj∈EEj+∑Jj∈Tγj =αCk+β∑j=1kaj1+bk−j[1+bm−1]=αCk+β22 +∑j=k+1k+maj1+bk+m−j  +θ∑j=1k(C[k]−C[j])+∑Jj∈Tγ[j] =(α+kθ)Ck−θ∑j=1kCj  +β∑j=1kaj1+bk−j[1+bm−1]  +β∑j=k+1k+ma[j]1+bk+m−j+∑Jj∈Tγ[j] =(α+kθ)∑j=1kaj1+bk−j  +β∑j=1ka[j]1+bk−j[1+bm−1]  −θ∑j=1kaj∑i=0k−j1+bi+β∑j=k+1k+maj1+bk+m−j  +∑j=k+m+1nγ[j] =∑j=1kaj1+bk−j{(α+kθ)+β[1+bm−1]}  −θ∑j=1ka[j]∑i=0k−j1+bi+β∑j=k+1k+ma[j]1+bk+m−j  +∑j=k+m+1nγj =∑j=1k+mwjaj+∑j=k+m+1nγj.

**Corollary 6 .**

If *m* = *n* − *k*, then

(11) $$\begin{array}{c} F\left(d_{1},D,\pi ,n-k\right)=\overset{n}{\underset{j=1}{\sum }}w_{j}a_{\left[j\right]}, \end{array}$$

where

(12) $$\begin{array}{c} w_{j}=\left\{\begin{array}{cc} \left\{\left(\alpha +k\theta \right)+\beta \left[{\left(1+b\right)}^{m}-1\right]\right\}{\left(1+b\right)}^{k-j} & \\ \;-\theta \overset{k-j}{\underset{i=0}{\sum }}{\left(1+b\right)}^{i},\;\;\;j=1,2,\ldots ,k & \\ \beta {\left(1+b\right)}^{k+m-j},\;\;\;\;j=k+1,k+2,\ldots ,n. & \end{array}\right. \end{array}$$

Equation (11) can be viewed as the scalar product of two vectors, *w*_*j*_ and *a*_[*j*]_, respectively, (*j* = 1,…, *n*). It is well known (from Hardy et al. [22]) that (11) is minimized by sorting the elements of the *w*_*j*_ and *a*_[*j*]_ vectors in opposite orders. This procedure can be done in *O*(*n*log⁡*n*) time. We refer to this rule as the HLP rule in the rest of the paper.

**Theorem 7 .**

If the number of $\overset{-}{D}$ jobs is given, then the problem 1  ∣*P*_*j*_ = *a*_*j*_ + *bt*∣*αd*_1_ + *βD* + *θ*∑_*J*_*j*_∈*E*_ *E*_*j*_ + ∑_*J*_*j*_∈*T*_ *γ*_*j*_ can be formulated as an assignment problem.

**Proof**

We define *z*_*jr*_ as a 0/1 variable such that *z*_*jr*_ = 1 if job *J*_*j*_ is scheduled in position *r*, and *z*_*jr*_ = 0, otherwise. We can formulate the problem 1∣  *P*_*j*_ = *a*_*j*_ + *bt*∣*αd*_1_ + *βD* + *θ*∑_*J*_*j*_∈*E*_ *E*_*j*_ + ∑_*J*_*j*_∈*T*_ *γ*_*j*_ as the following assignment problem:

(13) APm            Min⁡        ∑j=1n ∑r=1nCjrmzjrSubject  to∑r=1nzjr=1, j=1,2,…,n∑j=1nzjr=1, r=1,2,…,nzjr=0  or  1, j,r=1,2,…,n,

where

(14) Cjrm=aj∑i=0k−r1+biα+kθ+β1+bm−11+bk−r  −θ∑i=0k−r1+bi, r=1,2,…,kajβ1+bk+m−r,  r=k+1,k+2,…,k+mγj,β1+bk+m−r,  r=k+m+1,…,n.

Therefore, based on the above analysis, we can obtain a polynomial algorithm for the problem 1∣*P*_*j*_ = *a*_*j*_ + *bt*∣*αd*_1_ + *βD* + *θ*∑_*J*_*j*_∈*E*_ *E*_*j*_ + ∑_*J*_*j*_∈*T*_ *γ*_*j*_.

**Algorithm 8 .**

 

*Step 0*. By Lemma 4, calculate *k* = ⌈(*β* − *α*)/*θ*⌉.

*Step 1*. For *m* from 0 to *n* − *k* − 1, solve the above assignment problem AP(*m*) to obtain a local optimal schedule and the total cost *F*(*m*). 

*Step 2*. For *m* = *n* − *k*, first calculate the positional weights defined by (12) and assign the n jobs to the corresponding positions according to the HLP rule and then use (11) to evaluate the objective value *F*(*n* − *k*). 

*Step 3*. The global optimal schedule is the one with the minimum total cost given by min⁡{*F*(*m*)∣0 ≤ *m* ≤ *n* − *k*}.

Based on the above analysis, we have the following result.

**Theorem 9 .**

The scheduling problem 1∣*p*_*j*_ = *a*_*j*_ + *bt*∣*αd*_1_ + *βD* + *θ*∑_*J*_*j*_∈*E*_ *E*_*j*_ + ∑_*J*_*j*_∈*T*_ *γ*_*j*_ can be solved by Algorithm 8 in *O*(*n* ^4^) time.

**Proof**

For a given* m*, our problem becomes identical to the classical assignment problem and can be solved in *O*(*n* ^3^) time. Since 0 ≤ *m* ≤ *n* − *k* ≤ *n*, the overall time requirement of Algorithm 8 is *O*(*n* ^4^).

**Example 10 .**

Consider the instance with

(15) $$\begin{array}{c} n=5,\;\alpha =2,\;\beta =4,\;\theta =0.5,\;b=0.3,\\ a_{1}=4,\;a_{2}=3,\;a_{3}=6,\;a_{4}=9,\;a_{5}=11,\\ \gamma_{1}=6,\;\gamma_{2}=4,\;\gamma_{3}=5,\;\gamma_{4}=3,\;\gamma_{5}=30. \end{array}$$

Now we apply Algorithm 8 to solve Example 10. 

*Step 0.* Calculate the index *k* = ⌈(*β* − *α*)/*θ*⌉ = ⌈(4 − 2)/0.5⌉ = 4. 

*Step 1.* When *m* = 0, the *C*_*jr*_^0^ values can be calculated by (14) and given below:

(16) $$\begin{array}{c} C_{jr}^{0}=\left(\begin{array}{ccccc} 22.7780 & 19.0600 & 16.2000 & 14 & 6\\ 17.0835 & 14.2950 & 12.1500 & 10.5 & 4\\ 34.1670 & 28.5900 & 24.3000 & 21 & 5\\ 51.2505 & 42.8850 & 36.4500 & 31.5 & 3\\ 62.6395 & 52.4150 & 44.5500 & 38.5 & 30 \end{array}\right). \end{array}$$

The optimal job sequence is (*J*_2_, *J*_1_, *J*_3_, *J*_5_, *J*_4_).

The optimal objective value is *F*(0) = 101.9435. 

*Step 2.* When *m* = 1, the *w*_*j*_ values can be calculated by (12):

(17) $$\begin{array}{c} w_{1}=8.3309,\;\;w_{2}=6.7930,\;\;w_{3}=5.6100,\\ w_{4}=4.7000,\;\;w_{5}=4.0000. \end{array}$$

The optimal job sequence is (*J*_2_, *J*_1_, *J*_3_, *J*_4_, *J*_5_).

The optimal objective value is *F*(1) = 172.1247. 

*Step 3.* The global optimal objective is min{*F*(0), *F*(1)} = 101.9435. The global optimal schedule is (*J*_2_, *J*_1_, *J*_3_, *J*_5_, *J*_4_).

### 3.2. Job-Position-Dependent Learning Effect Model

By the same way as in the previous subsection, we consider the following scheduling problem: 1∣*P*_*j*_ = *a*_*j*_ *r* ^*a*^∣*αd*_1_ + *βD* + *θ*∑_*J*_*j*_∈*E*_ *E*_*j*_ + ∑_*J*_*j*_∈*T*_ *γ*_*j*_.

**Lemma 11 .**

For a given schedule *π* = (*J*_[1]_, *J*_[2]_,…, *J*_[*n*]_), if the starting time of the first job is 0, then *C*_[*r*]_ = ∑_*j*=1_^*r*^ *a*_[*j*]_ *j* ^*a*^ and ∑_*j*=1_^*n*^ *C*_*j*_ = ∑_*j*=1_^*n*^ *a*_[*j*]_(*n* + 1 − *j*)*j* ^*a*^.

**Lemma 12 .**

For the problem 1∣*p*_*j*_ = *a*_*j*_ *r* ^*a*^∣*αd*_1_ + *βD* + *θ*∑_*J*_*j*_∈*E*_ *E*_*j*_ + ∑_*J*_*j*_∈*T*_ *γ*_*j*_, if the job sequence is *π* = (*J*_[1]_, *J*_[2]_,…, *J*_[*n*]_) and $m=|\overset{-}{D}|$, then the objective function can be expressed as

(18) $$\begin{array}{c} F\left(d_{1},D,\pi ,m\right)=\overset{k+m}{\underset{j=1}{\sum }}{\bar{w}}_{j}a_{\left[j\right]}+\overset{n}{\underset{j=k+m+1}{\sum }}\gamma_{\left[j\right]}, \end{array}$$

where ${\bar{w}}_{j}=\left\{\begin{array}{c} (\alpha -\theta +\theta j)j^{a},\\ \beta j^{a} \end{array}\right.\begin{array}{c} j\;=\;1,2,\ldots ,k;\\ j\;=\;k+1,k+2,\ldots ,k+m. \end{array}$

**Proof**

By Lemmas 3 and 11, we have

(19) Fd,D,π,m =αd1+βD+θ∑Jj∈EEj+∑Jj∈Tγj =αCk+β∑j=k+1k+maj1+bn−j  +θ∑j=1k(C[k]−C[j])+∑Jj∈Tγ[j] =(α+kθ)Ck−θ∑j=1kCj+β∑j=k+1k+maj1+bn−j  +∑Jj∈Tγ[j] =(α+kθ)∑j=1kajja−θ∑j=1kaj(k+1−j)ja  +β∑j=k+1k+ma[j]ja+∑j=k+m+1nγ[j] =∑j=1k+mwjaj+∑j=k+m+1nγj.

**Corollary 13 .**

If *m* = *n* − *k*, then

(20) $$\begin{array}{c} F\left(d_{1},D,\pi ,m\right)=\overset{n}{\underset{j=1}{\sum }}{\bar{w}}_{j}a_{\left[j\right]}, \end{array}$$

where ${\bar{w}}_{j}=\left\{\begin{array}{c} (\alpha -\theta +\theta j)j^{a},\\ \beta j^{a} \end{array}\right.\begin{array}{c} \;j\;=\;1,2,\ldots ,k;\\ j\;=\;k+1,\;\;k+2,\ldots ,k+m. \end{array}$

Equation (20) can be viewed as the scalar product of two vectors, ${\bar{w}}_{j}$ and *a*_[*j*]_ vectors, respectively. The procedure can be done in *O*(*n*log⁡*n*) time by the HLP rule.

**Theorem 14 .**

If we fix the number of $\overset{-}{D}$ jobs, then the problem 1∣*p*_*j*_ = *a*_*j*_ *r* ^*a*^∣*αd*_1_ + *βD* + *θ*∑_*J*_*j*_∈*E*_ *E*_*j*_ + ∑_*J*_*j*_∈*T*_ *γ*_*j*_ can be formulated as an assignment problem.

**Proof**

It is similar to the proof of Theorem 7.Again, we can define

(21) $$\begin{array}{c} {\overset{-}{C}}_{jr}^{m}=\left\{\begin{array}{cc} \left(\alpha -\theta -\theta r\right)r^{a}a_{j}, & r=1,2,\ldots ,k\\ \beta r^{a}a_{j}, & r=k+1,k+2,\ldots ,k+m\\ \gamma_{j}, & r=k+m+1,\ldots ,n, \end{array}\right. \end{array}$$

as the cost of assigning job *J*_*j*_ (*j* = 1,2,…, *n*) to the *r*th (*r* = 1,2,…, *n*) position in the schedule. Then the problem 1∣*p*_*j*_ = *a*_*j*_ *r* ^*a*^∣*αd*_1_ + *βD* + *θ*∑_*J*_*j*_∈*E*_ *E*_*j*_ + ∑_*J*_*j*_∈*T*_ *γ*_*j*_ can be formulated as the following assignment problem:

(22) APm          Min⁡        ∑j=1n ∑r=1nC−jrmzjrSubject  to∑r=1nzjr=1, j=1,2,…,n∑j=1nzjr=1, r=1,2,…,nzjr=0  or  1, j,r=1,2,…,n.

Similar to Section 3.1, we have the following theorem.

**Theorem 15 .**

The scheduling problem 1∣*p*_*j*_ = *a*_*j*_ *r* ^*a*^∣*αd*_1_ + *βD* + *θ*∑_*J*_*j*_∈*E*_ *E*_*j*_ + ∑_*J*_*j*_∈*T*_ *γ*_*j*_ can be solved in *O*(*n* ^4^) time.

## 4. Conclusions

We have considered the single machine due-window assignment scheduling problem with variable job processing times. The objective is to minimize a linear combination of earliness, tardiness, the window location, window size, and weighted number of tardy jobs. We proposed a polynomial-time algorithm, respectively, for the learning effect and the deteriorating jobs. Obviously, if *a* > 0 (i.e., deterioration or aging effect) and *b* < 0 (i.e., shortening processing times), then the results of this paper still hold. In future research, we plan to explore more realistic settings, such as group technology scheduling problems, flexible flow shop scheduling problems, and unrelated parallel machines scheduling problems, or optimize other performance measures with variable job processing time.
